# Exploring a positive screen for eating disorders among 7,993 elite sport pupils in Sweden and its association with everyday food practices, demographics, and sports

**DOI:** 10.1186/s40337-026-01804-6

**Published:** 2026-10-23

**Authors:** Ida Davidsson, Agneta Hörnell, Carita Bengs, Magnus Ferry

**Affiliations:** 1 Department of Food, Nutrition and Culinary Science, Umeå University, Umeå, Sweden; 2 Department of Education, Umeå University, Umeå, Sweden

**Keywords:** Adolescents, Athletes, Eating disorders, Elite sport pupils, Food habits, Likely eating disorder, SCOFF, Sweden

## Abstract

**Background:**

Eating disorders can seriously affect athletes’ health and athletic performance. Negative consequences may also arise from suboptimal food practices and barriers in everyday life causing inadequate energy intake. Despite research about eating and health in various athletic populations, large-scale surveys of the Swedish elite sport school system have been lacking. Therefore, this study’s first aim was to explore Swedish elite sport pupils’ everyday food lives in terms of eating practices and perceived practical barriers to handling their daily food actions. A subset of these everyday eating practices and barriers was subsequently explored in relation to a positive eating disorder screen to answer the second aim which was to assess the prevalence of screening positive for eating disorder, and different associated factors.

**Methods:**

A cross-sectional survey was conducted together with the Swedish Sports Confederation in spring 2023, where we had added 20 food-related questions including a screening tool for eating disorders, SCOFF, at the end of the main survey. Nearly 8,000 pupils across Sweden responded to both parts of the survey. Following descriptive and bivariate analyses, we performed a multiple binary logistic regression to assess associations between demographic-, food-, and sports-related variables and a positive eating disorder screen.

**Results:**

Results showed that 9.5% of the pupils had a positive eating disorder screen. The factors most strongly associated with a positive screen were being female (OR = 3.50); regularly skipping meals (OR = 2.31); having a vegetarian or vegan diet (OR = 1.87); using dietary supplements (OR = 1.69); having more practical barriers (OR = 1.28); and practising an individual sport (OR = 1.40). Perceived higher levels of knowledge about what to eat to optimize health was inversely associated with a positive eating disorder screen (OR = 0.73). Half of the pupils perceived at least one practical barrier to handling their daily food actions, typically lack of time.

**Conclusions:**

Our findings underscore the importance of better support to prevent, identify, and address problematic eating behaviours in the elite sport schools, as well as implementing time-saving efforts.

**Plain English summary:**

Eating disorders can harm athletes’ health and their performance in sports. Negative effects can also come from not eating enough food because of poor eating habits or practical difficulties with food and meals. Up until now, no large studies focusing on eating and health among pupils at Swedish upper secondary elite sport schools have been made. This survey studied eating habits, food-related difficulties, and used the SCOFF-instrument as a screening tool for likely eating disorders among nearly 8,000 pupils. Almost one in ten pupils showed signs that could suggest an eating disorder. This was more common among girls, pupils who often skipped meals, those who did not eat meat, those with more practical difficulties related to eating, and those doing individual sports. Around half of the pupils said they had at least one practical problem, most often not having enough time. The study shows that elite sports schools need to give better support so pupils can manage food and eating in a healthy way.

**Highlights:**

• Almost ten per cent (9.5%) of the Swedish elite sport pupils screened positive for EDs. The strongest factors associated with a positive ED screen were being of female sex, regularly skipping meals, and excluding meat from the diet.

• Half of the pupils (49.1%) experienced at least one practical barrier in their everyday food lives, commonly lack of time and cooking skills. A higher number of barriers also increased the likelihood of a positive ED screen.

• These findings highlight the need for support from the schools; to prioritize initiatives to prevent ED risk among the pupils, and that all school and sport personnel should be able to identify problematic eating behaviour. Finally, practical education and additional school meals free of charge can further help support the pupils.

**Supplementary Information:**

The online version contains supplementary material available at https://doi.org/10.1186/s40337-026-01804-6.

## Background

The field of sports nutrition has evolved in recent decades and the importance of adequate nutrition to ensure athletes’ health and athletic performance is now widely recognized. Numerous and more extensive dietary recommendations for young athletes have been developed [1–4], and severe health risks related to nutrition and intensive physical activity have been discerned [5]. Proper nutrition is important for athletes regardless of age; however, for youth athletes it is also crucial to support normal growth and development [1]. Therefore, this study uses the concept *everyday food lives*, which is built upon the term “everyday lives” and defined as pupils’ “day-to-day actions relating to food” [6]. Research into such actions is, to date, nearly non-existent in the context of Swedish elite sport schools, with the exception of one small 25-year-old study focusing on the nutritional intake in this pupil group [7].

A person’s long-term relationship with food in relation to sports training and body image is established during youth [1]. This is a period when novel food-related practices such as meat-free diets or dietary supplement consumption might also be embraced. A major hurdle to proper diets for athletes across ages is lack of time for preparing food, while factors such as budget constraints and insufficient cooking skills are specifically highlighted for youth athletes in Australia [8]. It is still poorly understood if similar barriers are present among Swedish elite sport pupils and their specific context. There is also limited insights into their everyday food-related practices such as eating a plant-based diet or consuming dietary supplements.

For athletes, the presence of disordered eating (DE) and eating disorders (EDs) may not only have consequences for health but also for their athletic performance [9]. This position statement describes eating behaviour among athletes as a continuum from optimized nutrition, followed by a span of DE, to medically diagnosed EDs; e.g., anorexia nervosa and bulimia nervosa [10]. DE refers to a wide range of problematic or abnormal eating behaviours that do not meet clinical diagnosis for an ED [9]. Both DE and ED have gained increased scientific attention within the field of sports.

It is more common for athletes to present with DE than ED [9]. These are two distinct constructs with different prevalence rates. However, as discussed in a review [11]; this is not always clear in research. DE and ED are common among both youth and adult athletes, with a (combined) DE/ED prevalence of 6–45% among females and 0–19% among males [11]. A recent systematic review and meta-analysis [12] reported a mean prevalence of 19% for self-reported DE across 177 studies using different instruments. The authors specifically noted that several countries, including Sweden, rarely provided prevalence data.

General guidelines emphasize early detection and treatment of ED to promote diagnosis [13]. ED screening tools can therefore be considered of great value to identify those at risk before clinical assessment. Although, it should be noted that a positive screening result is not equal to an ED diagnosis.

ED diagnosis is more prevalent among youth athletes compared to age-matched, non-athletic controls [14], and among females practising sports that stress leanness and/or a specific body weight compared to athletes in other sports [15]. Proposed associated factors for athletes include general as well as gender- and sport-specific factors [11]. It has also been suggested that engaging in sport may be related to some protective factors for EDs [16].

A DE/ED-related threat to health and athletic performance in athletes is low energy availability (LEA), and this medical condition is frequent among youth athletes [17]. LEA occurs when insufficient energy is consumed to support increased energy expenditure from physical activity. This is problematic as adequate energy intake is the foundation of an athlete’s diet [4]. Common causes of LEA in athletes are DE and ED although the condition can arise from a variety of factors [9]. In addition, not all with DE or ED suffer from LEA. Over time, LEA can lead to relative energy deficiency in sport (REDs), with negative health and performance outcomes such as impaired growth and development, weakened bone health, decreased muscle strength, reduced immunity, and decreased endurance performance [5].

### Study context

Around 11,600 youth athletes (approximately 16–19 years of age) are enrolled at upper secondary elite sport schools across Sweden [18]. The school sports system allows for advancement within both education and sports (i.e., dual careers), and the pupils study their sport in the school subject *Special Sports* [19]. Teaching in the subject should cover several areas, including nutrition. A number of sub-areas related to nutrition are stated in the syllabus, e.g., general nutrition, diet and hydration, and methods for documenting nutritional intake. The term *body ideal* is also included, while education about EDs is not mentioned, although research has suggested that education is the top method for preventing the onset of EDs [20].

Some schools—national elite sport schools (NES)—recruit pupils at the national level, while regional/local recruitment applies for the majority of Swedish sports schools—the certified elite sport schools (CES) [18]. Pupils in the 2022/2023 academic year attended, in total, 57 NES and 479 CES, as compiled by documents [21]. In both types of schools, the pupils often need to move away from home and therefore manage their everyday food lives themselves already in adolescence. However, some degree of food-related support is often available. Free and nutritious school lunches are served in compulsory school [22], and most Swedish upper secondary schools also serve school lunches free of charge for all pupils [23]. Some elite sport schools also offer additional meals beyond lunches.

Despite the importance of proper nutrition for youth athletes the area is under researched in the present context. Thus, this study’s first aim was to explore Swedish elite sport pupils’ everyday food lives in terms of eating practices and perceived practical barriers to handling their daily food actions. A subset of these everyday eating practices and barriers was subsequently explored in relation to a positive eating disorder screen to answer the second aim which was to assess the prevalence of screening positive for eating disorders, and different associated factors.

## Methods

### Design and study procedures

This cross-sectional study was based on an online survey addressed to Swedish elite sport pupils in upper secondary school years 1–4 (approximately 16–19 years of age). The survey was conducted during the first half of 2023 as part of the Swedish Sports Confederation’s annual national survey exploring the pupils’ satisfaction with their sport and school environment, to which the study authors added 20 supplementary questions centred around food and nutrition. The focus areas included eating practices, dietary supplement use, food support, nutrition information sources, nutrition education, and ED. With respect to ED screening, the validated instrument SCOFF (Sick, Control, One stone [6 kg], Fat, Food) [24] was employed. Development of the other questions, including the six practical food-related items, was based on previous literature combined with the researchers’ field knowledge. The initial questions were revised following comments by teachers and pupils in a pilot study at an elite sport upper secondary school in Sweden.

The number of registered participants for the annual survey was 10,145 and the response rate was 89% (*n* = 9,060). This study draws on the 7,993 pupils who completed the survey and also answered the supplementary questions about food and nutrition, i.e., the nutrition sub-study. A more detailed description of the survey’s methods and the participants can be found in (submitted manuscript, Author et al.).

### Survey questions

The following research areas were covered through single-choice, multiple-choice, and Likert-scale questions.

#### Demographics

Sex was reported as male/female/other or don’t want to state. Only male (0) vs. female [1] was used in the analysis due to the small number of other/decline to state responses (*n* = 23). Accommodation was categorized into parental home, pupil residence, and other (i.e., own apartment, shared apartment with friend(s), roomer, and other accommodation).

#### Sports-related variables

Type of sport (*n* = 47) was dichotomized as team sports (0) (American football, bandy, basketball, curling, floorball, handball, hockey, rugby, soccer, team gymnastics, and volleyball vs. individual sports [1] (all others). Favourable physical requirements for sports were categorized by the authors as: “Big and strong” (American football, ice hockey); “Small and slim” (athletics, biathlon, cross-country skiing, cycling, diving, figure skating, gymnastics, orienteering, ski orienteering, team gym, triathlon); “Weight-class divided” (boxing, judo, karate, powerlifting, taekwondo, wrestling); and “Neutral” (all others).

#### Variables on dietary practices

Types of diet were reported as omnivore (in the survey explicitly defined as “eating most foods including fish and meat”); vegetarian (“not eating meat and fish but egg and/or milk”), and vegan (“eating a completely plant-based diet and excluding meat, fish, egg and milk”). In inferential analysis, omnivore (0) vs. non-meat eater [1] (vegetarian and vegans pooled together) was used due to the small number of vegans (*n* = 13). Dietary supplement use was merged and coded as no (0) = not consuming energy supplements (e.g., protein powder, bars, gels, carbohydrate drinks, gainers, etc.), or performance-enhancing supplements (e.g., pre-workout supplements, creatine, caffeine tablets, nitrate, bicarbonate, beta-alanine), and yes [1] = at least one of these types. Main meals (breakfast, lunch, dinner) were reported yes vs. no for each meal type. In inferential analysis, these were pooled into not eating (0) vs. eating [1] three main meals daily. The number of in-between meals usually eaten was reported as 0 to 5 or more. When more than one option was chosen (e.g., 2, 4 and 5 in-between meals), the median numbers were calculated. The pupils were asked to rate “I know what to eat to optimize my health” and “…my performance”, respectively, on a scale from 1 (“strongly disagree”) to 5 (“strongly agree”). The “don’t know” answers were excluded. Six practical food-related barriers when manoeuvring one’s everyday food life were reported as no (0) vs. yes [1]. Barriers comprised lack of: time to cook; time to plan/shop for food; cooking skills; money to eat good and sufficient food; cooking facilities; and storage facilities. A summed index variable (0–6) was also generated. Whether teaching about eating disorders had been addressed in the subject *Special Sports* was reported as no (0) vs. yes [1].

#### Eating disorders

The validated five-item screening tool SCOFF was employed to flag pupils with a positive ED screen [24], following a Swedish translation [25]. The choice of SCOFF was mainly based on its brevity, in combination with its high sensitivity and specificity [24]. This generic instrument is often used in studies of athletes and has also been found to be valid in this population [26]. The original instrument includes five separate yes/no questions. In our study, these were changed into statements and merged into one multiple-response question, e.g., the original question “Do you worry you have lost control over how much you eat?” (yes/no) was changed to “I worry I have lost control over how much I eat” (followed by a box to tick only if they agreed with the statement). This modified format was used as we could only include 20 food-related questions in the main survey, and it was not validated for our study. Two additional statements were included in the multiple-response question: “I worry that some among my peers experience some of the mentioned issues” and “None of the mentioned issues are true for me” (e.g., not agreeing with any of the statements for oneself or for peers). Answering “yes” to two or more of the five SCOFF questions is seen as a positive screen, suggesting that an ED may likely be present and that clinical ED assessment is needed [24]. The five variables were re-coded into a binary variable, under cutoff (0) vs. over cutoff [1].

Many scholars have used and interpreted SCOFF as a measure of DE (cf. 12). This includes a Swedish validation study of a general adolescent population [27]. The authors suggested that only one “yes” to the five questions might be sufficient for a positive DE screen in this population. Based on this paper, we therefore also constructed a binary variable with the lower cutoff of one or more ticked statements, specified a priori. This variable was only used for additional descriptive analysis.

### Data analysis

Categorical variables were summarized as frequencies and percentages, and continuous variables as means (± SD), and as median (p25; p75) when data distribution was skewed (i.e., for the variable regarding number of practical barriers). Mean values are, however, given for SCOFF to facilitate comparisons with other studies even if skewed. A significance level of *p* < 0.01 was chosen given the large sample size, i.e., to not overemphasize minimal or “practical unimportant” differences. A range of analyses were conducted guided by statistical guidelines [28, 29]. Independent samples t-test, Mann-Whitney U test and chi-square test of independence were employed for analysis between groups. For chi-square tables larger than 2 × 2, adjusted residual analysis was performed with a 1% significance level (AR < -2.58 or > + 2.58). Strengths of relationships were interpreted using the effect sizes: phi coefficient (Φ), Cramer’s V (w), Cohen’s d (d) and (*r*). General cutoffs were considered [30, 31], and supplemented with the letters L (large), M (medium) and S (small), regardless of effect size employed. Additionally, VS (very small) was used for effect sizes below the threshold for a small effect size.

Bivariate analysis and multiple binary logistic regression were performed with the dependent variable set as SCOFF screening under cutoff (0) vs. over cutoff [1]. The independent variables were chosen a priori based on the study aims, previous research and theoretical relevance. Bivariate analyses were used to provide a descriptive overview of associations, and not as a variable selection procedure. Excluding variables with non-significant bivariate associations might have led to the exclusion of potential confounders. The multiple logistic regression was performed using the forced entry method [29] to simultaneously examine factors associated with a positive ED screen while adjusting for all other variables in the model. Multicollinearity testing was performed between all independent variables. Presence of substantial multicollinearity was ruled out as all tests resulted in tolerance > 0.1 and variance inflation factor (VIF) values < 10 (commonly used cutoffs according to [29]. The presented regression model was tested against baseline model performance using the omnibus test and the Hosmer–Lemeshow test to assess goodness of fit [28, 29]. No causal interpretation between the associated factors and the dependent variable was possible due to the cross-sectional design of the study, i.e., the associations are only statistical. The analyses were performed using IBM SPSS Statistics 28.0. For both the inferential and multiple logistic regression analysis, we omitted participants with any missing data. Proportions of missing data were minor (0–3%), and the exact number of pupils included in each comparison test have therefore not been written out in the results.

### AI disclosure

Microsoft 365 Copilot was used for minor language editing to improve the clarity of the text.

## Results

First, a description of the characteristics of the pupils and their everyday food practices is provided. Next, the prevalence of a positive ED screen amongst them and associated factors are presented.

### Sample characteristics and everyday food practices

In the study sample (*n* = 7,993), more than half were males (59.2%), 40.3% females (Table 1), and 0.3% other/did not want to state. Most pupils lived in their parental home (60.3%) and practised a team sport (67.5%). The majority were active in sports favouring no special physical requirement, e.g., “neutral” (80.4%), 15.3% in sports categorized as favouring a “small and slim” physique, 2.9% a “big and strong” physique, and 1.4% practised a “weight-class divided” sport.

**Table 1:** Characteristics of the pupils and a positive ED screen, stratified by characteristics

		n	%	Over cutoff ≥ 2 yes (%)	p-value effect size^a^
Biological sex	Female	3224^b^	40.3	15.8	**p < 0.001** Φ = 0.18/S
	Male	4728^b^	59.2	5.1	
Accommodation	Parental home	4822^b^	60.3	9.0	*p* = 0.193
	Pupil residence	704^b^	8.8	10.5	
	Other	2446 ^b^	30.6	10.1	
Dietary pattern	Omnivore	7851	98.2	9.2	**p < 0.001** Φ = 0.057/VS
	Vegetarian/vegan	142	1.8	21.8	
Dietary supplement use^c^	No	4857	60.8	8.6	**p < 0.001** Φ = 0.038/VS
	Yes	3136	39.2	10.8	
Three main meals daily	Yes	6751	84.5	8.1	**p < 0.001** Φ = 0.108/S
	No	1242	15.5	16.8	
Favourable physical requirement	Big and strong	233	2.9	4.3	*p* = 0.019
	Small and slim	1220	15.3	10.7	
	Weight-class divided	112	1.4	11.6	
	Neutral	6428	80.4	9.4	
Type of sport	Team ball	5395	67.5	8.4	**p < 0.001** Φ = 0.051/VS
	Individual	2598	32.5	11.6	
Eating disorders have been addressed in the teaching	Yes	2019	25.3	9.6	*p* = 0.807
	No	5974	74.7	9.4	

^a^ Chi-square test of Independence, and phi coefficient for effect size. Significance level p<0.01 (significant results marked in bold)

^b^ Numbers differ due to missing data or excluded response options in the analysis

^c^Energy supplements and/or performance-enhancing supplements

Most pupils reported being omnivores (Table 1), and plant-based diets were rare: vegetarian (1.6%) and vegan (0.2%). Plant-based diets, in total, were more common among females than males (3.0% vs. 0.9%; *p* < 0.001, Φ = 0.079/VS). Among all, 15.5% did *not* eat three main meals daily, with no significant difference between sexes or accommodation types. Breakfast was the most skipped meal, usually *not* eaten by 14.1% of the pupils. Almost all reported that they usually ate at least one in-between meal (96.4%), 38.2% two in-between meals, and 17.7% reported a higher frequency. Almost four in ten reported consuming dietary supplements (energy- and/or performance-enhancing types) (Table 1). Consumption was more prevalent among males than females (45.9% vs. 29.5%; *p* < 0.001, Φ = 0.165/S).

The pupils agreed strongly on a five-point Likert scale that they knew what to eat to optimize (i) *their performance* (4.0 ± 0.90), and (ii) *their health* (4.1 ± 0.88). Males agreed more strongly than females on knowing what to eat to optimize their health (Table 2). When asked to report on barriers that impacted their possibilities to practically manoeuvre their everyday food lives, the most frequently perceived barriers were related to lack of: time to cook (26.5%); time to plan/shop for food (23.5%); cooking skills (14.0%); and money to eat good and sufficient food (9.8%). Fewer pupils reported a lack of storage (2.3%) and cooking facilities (2.1%). Reporting lack of time differed partly between the three accommodation types. No difference was found in relation to time to cook, but for time to plan/shop for food (*p* < 0.001, w = 0.044/VS), those living in “other accommodation” were more likely to report this barrier (AR = 4.0) and those living in parental homes less likely (AR= -3.3). When all barriers were considered together, almost every second pupil (49.1%) reported that they experienced at least one of them. This was more common among females than males (51.3% vs. 47.5%; *p* < 0.001, Φ = 0.038/VS). Experiencing two or more barriers was reported by 22% overall and was more common among females than males (25.3% vs. 19.6%; *p* < 0.001, Φ = 0.067/VS). The most typical combination of reporting two barriers was a lack of time to plan/shop for food and to cook.

**Table 2:** Comparisons of continuous variables between a positive vs. negative ED screen, and between biological sexes

	*SCOFF (n = 7* *993)*			*Biological sexes (n = 7952)*		
	Under cutoff < 2 yes	Over cutoff ≥ 2 yes	*p*-value effect size^a^	Males **(n = 4728)**	Females **(n = 3224)**	p-value effect size^a^
“I know what to eat to optimize my health”^b^, m ± SD	4.1 ± 0.86 (*n*=7053^c^)	3.8 ± 0.97 (*n*=742^c^)	**p** **< 0.001** d = 0.426/S	4.2 ± 0.84 (4591^c^)	4.0 ± 0.91 (3163^c^)	**p** **< 0.001** d = 0.234/S
Practical barriers (0–6), md (p25; p75)	0 (0; 1)^d^ (n = 7236)	1 (0; 2)^d^ (n = 757)	**p** **< 0.001** r= -0.096/VS	0 (0; 1)^d^	1 (0; 2)^d^	**p** **< 0.001** r=-0.072/VS

^a^Independent samples t-test and Mann–Whitney U test; effect sizes: Cohen’s D and r. Significance level p<0.01 (significant results marked in bold)

^b^Measured on a five-point Likert rating scale spanning from 1 (“strongly disagree”) to 5 (“strongly agree”)

^c^Numbers differ due to excluded response options in the analysis

^d^Mean ranks: 3931.2 vs 4625.8; 3848.9 vs 4163.6

### Eating disorders

In total, 9.5% (females: 15.8%; males: 5.1%) had a positive ED screen. The sum of pupil’s agreements to the five questions included in SCOFF are displayed in Table 3, in total and stratified by sex. Due to the large proportion of “zero-reporters”, the data was skewed, resulting in a mean score of 0.4 for all, and 1.5 for those who reported at least one “yes”.

**Table 3:** The pupils’ sum of agreements to the five questions included in SCOFF

Number of agreements	Total (*n* = 7993)	Males (*n* = 4728)^a^	Females (*n* = 3224)^a^
0	74.7	81.6	64.7
1	15.8	13.3	19.5
2	6.3	3.5	10.5
3	2.4	1.3	3.8
4	0.6	0.2	1.1
5	0.2	0.1	0.4
Total mean ± SD	0.4 ± 0.79	0.3 ± 0.62	0.6 ± 0.95
Mean ± SD for pupils with 1–5 agreements	1.5 ± 0.83	1.4 ± 0.74	1.7 ± 0.88

Data is presented as percentage of pupils for each agreement sum and as mean ± SD, in total and stratified by sex^a^

^a^“Other/don’t want to state” (n=23) are excluded; missing data on sex (n=18)

**Fig. 1:**
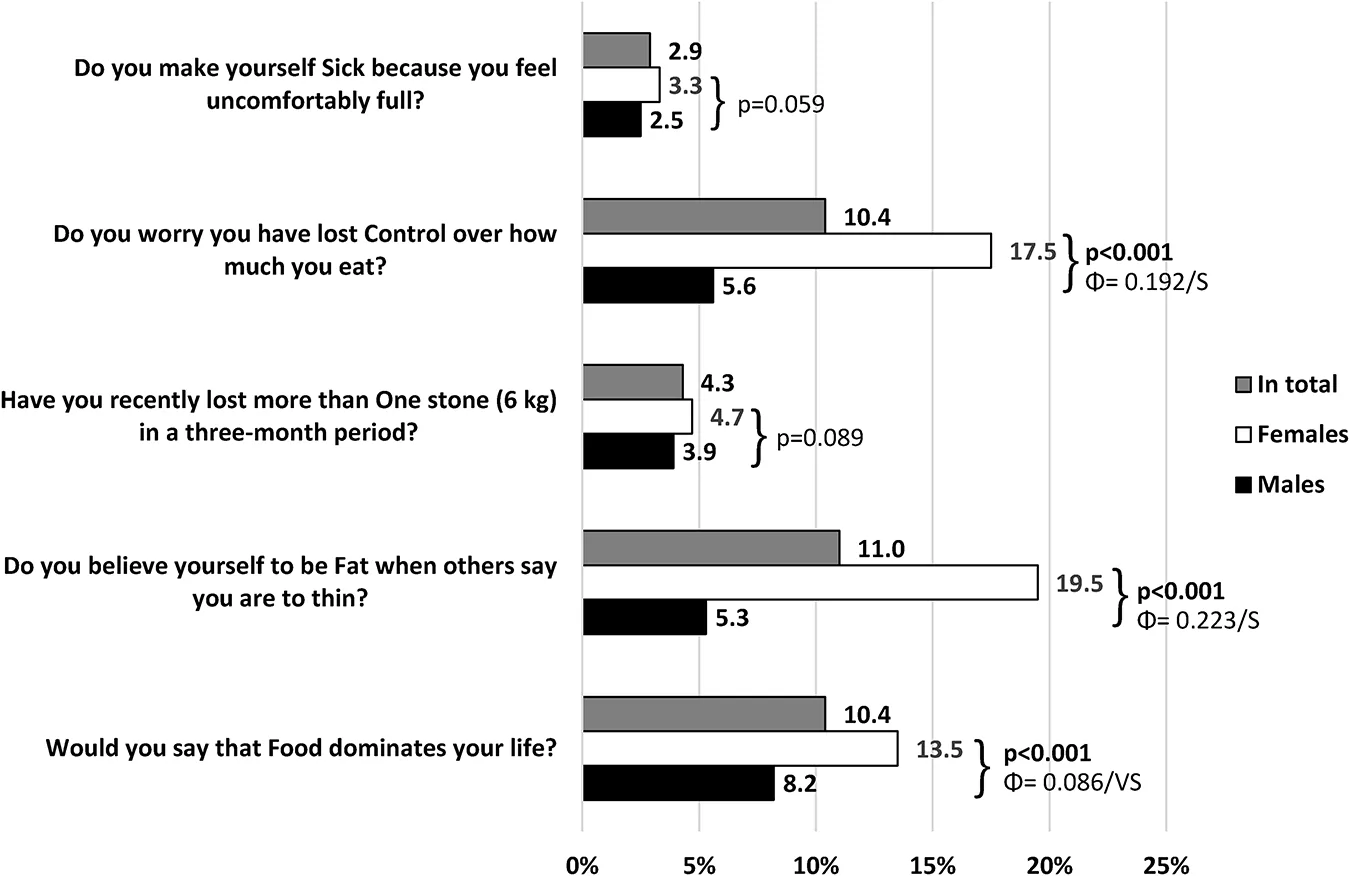
Pupils (*n* = 7993; females = 3224, males = 4728) agreeing to the five questions included in SCOFF [24]. Bars are sorted in line with the SCOFF acronym. Significance testing between biological sexes: chi-squared test of independence; effect size: phi coefficient

Figure 1 shows the proportion of pupils who agreed to the five questions included in SCOFF. Three of the questions received almost equal numbers of agreement overall. However, more females than males agreed to each of these questions. No such differences were found for the two other questions (where the proportion of responders who agreed was lower). The pupils were also asked if they were worried that other pupils experienced any of the issues addressed in SCOFF, and 12.3% agreed. We also explored a cutoff of a single agreement to the five SCOFF questions as a positive DE screen, based on a suggestion from a Swedish validation study [27], although the authors stated that further validation is needed. Using this exploratory cutoff, 25.3% screened positive for DE (females: 35.2%; males 18.4%).

Most variables were in bivariate analysis associated with a positive ED screen (≥ 2 agreements on SCOFF) (Tables 1 and 2). However, there was no significant association between a positive ED screen and type of accommodation or whether education about eating disorders had been provided (*p* = 0.193 and *p* = 0.807, respectively). Neither was the favourable physical requirement type for the sport the pupils were engaged in associated with screening positive (*p* = 0.019). Pupils with the favourable physical requirement “big and strong” were in percentage terms less likely to present a positive ED screen (4.3% did so) compared to the rest. The largest proportion of positive ED screeners were those categorized within “weight-class divided” (11.6%) followed by “small and slim” (10.7%).

The same seven variables that were associated with a positive ED screen in bivariate analyses also contributed significantly to the multiple logistic regression model (Table 4). The strongest association between a positive ED screen was being of female sex (OR = 3.50), followed by three diet-related associations: *not* eating three main meals daily (OR = 2.31), being a non-meat eater (OR = 1.87), and using dietary supplement(s) (OR = 1.69). A positive ED screen was also associated with practising an individual sport (OR = 1.40). Furthermore, for each additional practical barrier the pupils reported, the odds of a positive screen increased by around 28% (OR = 1.28), and for each higher number of agreements on the rating question “knowing what to eat to optimize my health”, the odds of a positive ED screen decreased by approximately 27% (OR = 0.73).

**Table 4:** Multiple logistic regression of the association between independent variables and a positive ED screen (n= 7739)

			99% C.I.	
	Sig.	OR	Lower	Upper
Sex (0 = male)	**< 0.001**	3.50	2.79	4.39
Three main meals daily (0 = yes)	**< 0.001**	2.31	1.80	2.95
Types of diet (0 = omnivore)	**0.005**	1.87	1.05	3.31
Dietary supplement use^a^ (0 = no)	**< 0.001**	1.69	1.37	2.10
Practical barriers (0–6)	**< 0.001**	1.28	1.16	1.41
Type of sport (0 = team sport)	**0.003**	1.40	1.05	1.86
“I know what to eat to optimize my health” (1–5)^b^	**< 0.001**	0.73	0.65	0.81
Eating disorders have been addressed in the teaching	0.210	1.12	0.88	1.43
Favourable physical requirement^c^ (ref. big and strong)		1.00		
Small and slim	0.626	1.20	0.45	3.19
Weight-class divided	0.931	1,04	0.30	3.58
Neutral	0.321	1.42	0.57	3.5
Accommodation^c^ (ref. parental home)		1.00		
Pupil residence	0.493	0.90	0.61	1.33
Other	0.938	0.99	0.78	1.27

Positive ED screen= ≥2 “yes” on the five SCOFF questions

Nagelkerkes R2 = 0.133, Hosmer–Lemeshow test=0.155 (ref: >0.05), omnibus test <0.001 (ref: <0.05)

^a^Energy supplements and/or performance-enhancing supplements

^b^“Measured on a Likert rating scale spanning from 1 (“strongly disagree”) to 5 (“strongly agree”)

^c^Overall p-values: p=0.383 vs p=0.784

## Discussion

Our findings show that the elite sport pupils reported to a high extent that they knew what to eat to optimize their health and athletic performance, i.e., perceived that they had good knowledge about sports nutrition. However, we also identified several factors that was associated with a positive ED screen, potentially also LEA or otherwise harmful behaviour.

Almost ten per cent of the pupils had a positive ED screen. To the best of our knowledge, this is the first large-scale ED screening estimate provided for this population. Our results showed that the prevalence for a positive ED screen on SCOFF was higher among females than males. Although the employed instruments and study outcomes differed, this is in line with findings from two international reviews; one that reported combined DE/ED rates [11], the other self-reported DE prevalence (including SCOFF) [12]. These reviews draw attention to several challenges with prevalence comparisons. SCOFF is often used to measure DE, although the instrument is designed and validated as an ED screening tool (as used in the present study) [32]. The continuum of eating behaviours from optimized nutrition, via DE to ED as described by Wells et al. [9] can make the area difficult to study. Indeed, in an overview of ED among athletes [11] it is stated that comparisons between studies are complicated due to the variety of assessment tools, different definitions, and inconsistent cutoffs used in sport-related research.

As far as we know, the validity and reliability of SCOFF for Swedish youth athletes have not been studied. However, Danish researchers [26] validated SCOFF against ED diagnostic interviews among athletes (age span 16–47 years; mean age 23 years) and found this generic instrument to be valid as an ED screening tool (sensitivity: 94%; specificity: 88%). In a Swedish general adolescent population, an increase in concerns was observed when SCOFF was validated to screen for DE (in their paper called “disordered eating behaviours”) against the Eating Disorder Examination Questionnaire (EDE-Q) [27]. Although the researchers found SCOFF to be a fairly good measure of DE, they argued that the requirement of two “yes” on the five SCOFF questions might risks missing DE, in particular among adolescent boys. They therefore suggested that one “yes” could be sufficient, as it could increase early detection. A recent systematic review mentions the importance of early detection but also highlight that caution is needed when SCOFF is used to assess DE as the instrument is not robustly validated for this [32].

When we used SCOFF to screen for ED according to the original instrument [24], we found a prevalence of 9.5%. When we used the approach by Hansson et al. [27], with the low cutoff of one “yes”, we found that a much larger proportion of the pupils, every fourth pupil (females: 35.2%; males 18.4%) had screened positive for DE, which would indicate a need for follow-up on these pupils. Taken together, we see a need for a validation study of SCOFF among Swedish youth athletes, which would be needed to assess the validity of our results. Such a study would benefit from placing specific emphasis on whether ED or DE screening should serve as the most appropriate outcome measure.

In total, seven variables were significantly associated with a positive ED screen, most of them aligning with earlier suggested associated factors (and risk factors) in the DE/ED field [9, 12, 20]. Many additional influencing factors have also been proposed in the literature, suggesting a multifactorial nature of EDs among athletes. Surprisingly, we found no significant association between the favourable physical requirement of one’s sport (“big and strong”/“small and slim”/“weight-class divided”/“neutral”) and a positive ED screen (*p* = 0.019). This was unexpected, as earlier studies have highlighted that athletes practising weight-divided sports, and sports that stress leanness or a high power-to-weight ratio are most at risk for DE [33], which could indicate an increased risk for ED. However, no difference was found between the prevalence of symptoms associated with ED and female athletes in weight-sensitive vs. less weight-sensitive sports at Norwegian upper secondary elite sport schools [14]. Potential reasons for the inconsistency may be the use of different screening and diagnostic instruments, different categorizations of groups, or underreporting in some groups, which highlights the need for further research.

To prevent the onset of EDs in athletes, education is suggested to be mandatory for all coaches and athletes regardless of sport [20]. The proposed educational strategy is to enhance knowledge about the highest risk groups, associated risk factors, and strategies for early identification. Educational initiatives should be tailored to the sports and athlete group (e.g., young people). This was not supported by our results as no significant association was found between a positive ED screen and whether pupils acknowledged that teaching about EDs had been addressed in the subject of *Special Sports*. However, we did not study the extent of such teaching, nor what was included in the teaching. Our result should therefore be interpreted with caution, and further research is needed.

Only 25.3% of the pupils reported that EDs had been addressed in the teaching. As other critical aspects of DE and ED prevention endorse de-emphasizing body weight and advocating health and well-being [33], teaching related to EDs (or DE) might have been provided for more elite sport pupils than reported, although not explicitly. With respect to young female pupils, Martinsen et al. [34] presented promising results using a one-year ED prevention program in Norwegian elite sport high schools. Their multi-targeted program (pupils/parents/coaches inside and outside school) focused specifically on improving self-esteem and self-efficacy. The coaches and parents also engaged in learning materials about detecting and managing DE and ED.

It is important that individuals around the pupils are aware of signs of DE, as athletes seldom self-identify [20]. The Swedish Sports Confederation’s guidelines [35] on food and nutrition encourage the sports movement to not only pay attention to signs, but also to address their concerns to the athlete and their parents and to contact a specialist, e.g., physician, nutritionist or dietitian. In the present context, this responsibility rests upon all school personnel (school leaders, teachers, and coaches). Talking about EDs in a sports environment might, however, be seen as a “taboo topic”, as recognized in a small qualitative study of Swedish elite sport pupils in lean sports [36]. Specifically, discussing this topic may pose challenges for elite coaches if they feel guilt for the athlete’s DE [33]. If recognized and addressed, counselling could be provided by the elite sport schools or the specific sports federations but may differ according to school or sport.

Although DE and ED are often the underlying cause of LEA in athletes, a lack of knowledge, time, and resources can also cause LEA [9]. Almost half of the elite sport pupils (49.1%) experienced at least one of the practical barriers we asked about, and thus many may be in the risk zone. The most-reported barriers were lack of time to plan/shop for food and to cook, aligning with international findings [8]. Elite sport pupils still living in their parental home were less likely to perceive lack of time to plan/shop for food. This can probably be explained by additional support from family members. By contrast, living in their parental home was not associated with eating more regular meals. Our finding that 15.5% of the pupils usually skipped at least one main meal is concerning because this behaviour may quickly lead to inadequate nutritional intake since athletes often have significantly elevated needs caused by high physical activity [37], i.e., it is hard to meet these high needs if few meals are eaten. It should, however, be noted that the proportion of “main meal-skippers” (15.5%) might be overestimated, as some pupils’ dietary practices may differ from those we studied (e.g., replacing “ordinary” breakfast with smaller in-between meals before and after training).

Following a plant-based dietary pattern was found to be one of the three factors most strongly associated with a positive ED screen. This supports evidence from a systematic review [38] among adolescents and young adults in a general population. Most included studies found significant associations between vegetarianism and EDs, both diagnosed or indicated through instruments. However, in accordance with our study they could not draw any conclusions about causality.

Although few pupils excluded all animal-based foods from their diet (1.8%), it can be assumed that diets with a high proportion of plant-based foods will increase as general nutritional recommendations progressively emphasize that a healthy diet is high in plant-based foods [39]. The choice of eating a plant-based diet can be driven by a multitude of reasons such as health-related, environmental or animal ethical motives; however, following a vegetarian diet can also stem from economic constraints or the masking of DE [4]. A consensus statement [9] noted that a behavioural change towards restrictive eating such as cutting out food groups (e.g., eating only plant-based) is a warning sign for DE and ED in athletes. In the light of our findings, specific attention should be given to the elite sport pupils that follow, or start following, a plant-based diet.

Besides the finding that dietary supplement use was a factor associated with a positive ED screen, additional risks associated with supplement consumption is also worth noting. Almost four in ten pupils reported using dietary supplements, although consumption for youth athletes to enhance performance is not recommended due to the lack of scientific evidence on possible long-term consequences, and because consumption exaggerates beliefs about supplements’ potential to enhance performance [1]. Some supplements are moreover harmful or might be so in combination or due to improper use [40].

Based on our findings, we suggest that increased support for the pupils is needed in three specific ways. First, the schools should prioritize initiatives to prevent ED risk among the pupils. Increased ED education could be a possible strategy to lower the risk, although our data were insufficient to provide empirical evidence in this matter. Secondly, the teachers and coaches ought to be educated in these areas to be able to identify risk behaviours and address them in a timely manner, especially as the pupils also report several practical barriers. Thirdly, the schools should strive to reduce time constraints in the pupils’ everyday food lives. One course of action could be to serve further free meals in addition to the school lunches that are most often provided in upper-secondary schools [23]. Increased practical education related to food and meal planning could also decrease some of the practical barriers to eating enough and nutritiously.

### Strengths and limitations

A key strength of this study was its inclusion of 7,993 participants, representing nearly 70% of eligible pupils. The study also provided the first large-scale estimates of ED screening rates and associated factors in the population. As we chose a more stringent significance level (*p* < 0.01) instead of the standard threshold (*p* < 0.05), the risk of type I errors was reduced. Although a lower significance level increases the risk for type II errors, we assessed this risk to be limited due to the large sample size. In addition, we presented effect sizes for the bivariate analysis to decrease the risk of overinterpreting the results.

However, there are some methodological limitations. Since data was self-reported, response biases such as socially desirable responses may have occurred. This might be especially true for the SCOFF questions as DE and ED can be especially sensitive issues. Another limitation to address is that we did not validate our modified SCOFF format, i.e. altering the original five separate yes/no questions to one multiple-response question. Hence, we cannot assess if the original cutoff is valid to our data. Further, Smyth et al. [41] observed that forced-choice formats (e.g., having to answer yes or no on a number of questions) led to significantly more options marked per question compared to check-all-that-apply formats; hence, the prevalence of positive ED screens among the pupils may be underestimated compared to studies using the original SCOFF. We might also have overlooked important aspects, e.g., of perceived practical barriers, due to closed-ended questions.

It should also be noted that no causal interpretation between the independent variables and the dependent was possible due to the cross-sectional design of the study. However, we believe that the logistic regression analyses, in combination with the bivariate analysis and effect sizes, are of great value and that the findings can form the basis for further exploration, preferably prospective studies as randomised controlled studies are impossible for ethical reasons.

## Conclusions

In this countrywide study among Swedish elite sport pupils, 9.5% had a positive ED screen. This represents the first estimate provided in the present population on a large scale. Seven factors associated with a positive ED screen were also found: being of female sex; regularly skipping meals; excluding meat from the diet; using dietary supplements; having more practical barriers; practising an individual sport; and perceived lower levels of knowledge about what to eat to optimize health. These findings add to the growing body of research aimed at preventing and identifying DE and ED among athletes. Additionally, half of the pupils (49.1%) experienced barriers in their everyday food lives, particularly lack of time to plan/shop and cook, and cooking skills. Further qualitative research is needed to explore barriers, and facilitators more thoroughly in a school context.

## Electronic Supplementary Material

Below is the link to the electronic supplementary material.

Figure 1

## Data Availability

The datasets generated and analyzed during the current study are not publicly available as further analyses and publications are ongoing but may be available from the corresponding author on reasonable request.

## Abbreviations

ED: Eating disorder
DE: Disordered eating
LEA: Low energy availability
REDs: Relative energy deficiency in sport
