# Supplementary material for: Exploring a positive screen for eating disorders among 7,993 elite sport pupils in Sweden and its association with everyday food practices, demographics, and sports
Source: J Eat Disord. 2026 Oct 23;14:250. doi: 10.1186/s40337-026-01804-6 (PMC13695266; doi:10.1186/s40337-026-01804-6)
Supplement: Supplementary file 1 [file 40337_2026_1804_MOESM1_ESM.pdf]

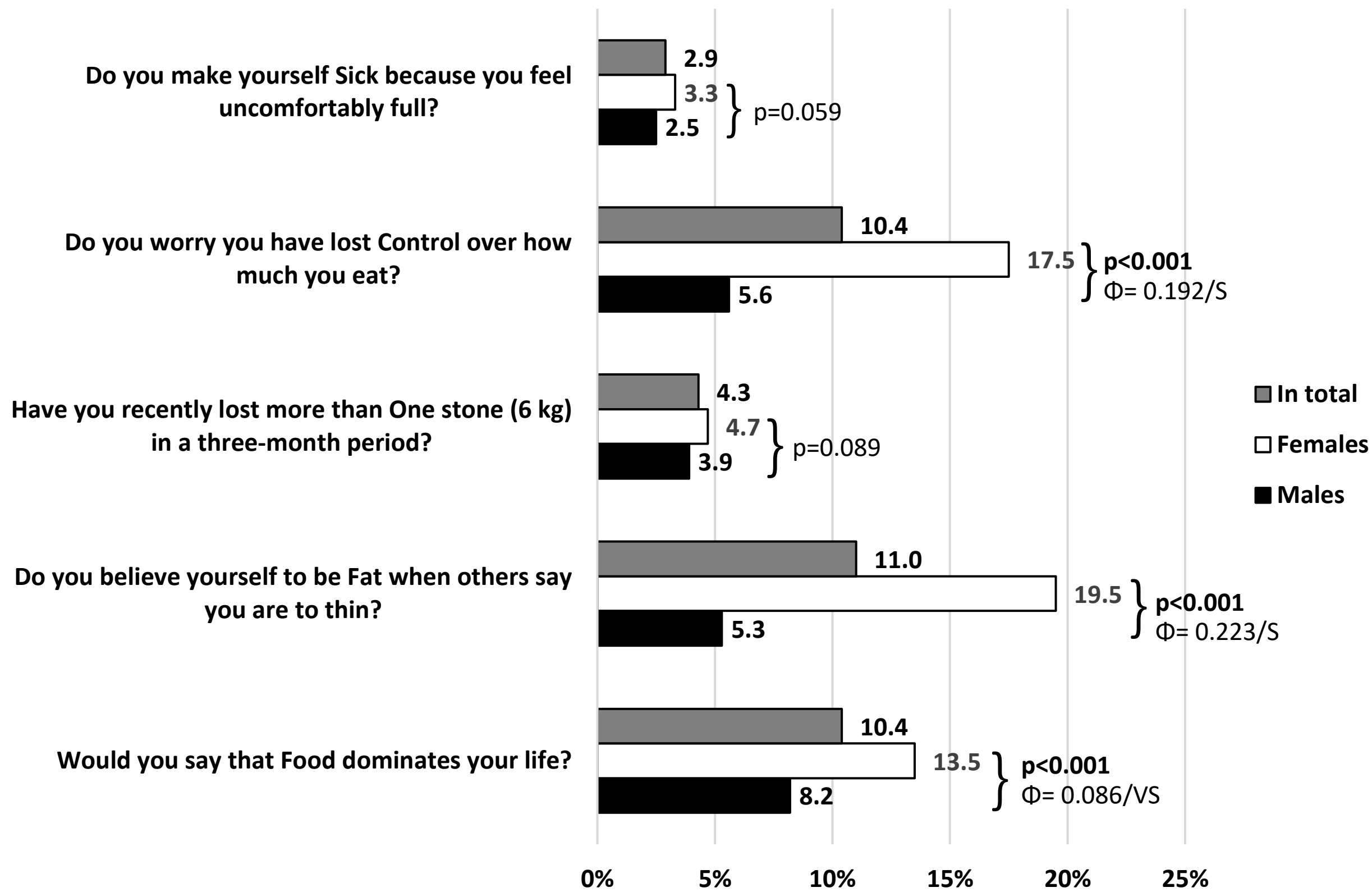

Do you make yourself Sick because you feel uncomfortably full?
2.9
3.3
2.5
p=0.059
Do you worry you have lost Control over how much you eat?
10.4
17.5
5.6
p<0.001
Φ= 0.192/S
Have you recently lost more than One stone (6 kg) in a three-month period?
4.3
4.7
3.9
p=0.089
Do you believe yourself to be Fat when others say you are to thin?
11.0
19.5
5.3
p<0.001
Φ= 0.223/S
Would you say that Food dominates your life?
10.4
13.5
8.2
p<0.001
Φ= 0.086/VS
In total
Females
Males
0%
5%
10%
15%
20%
25%
